# Hypertonic saline nasal irrigation and gargling for suspected or confirmed COVID-19: Pragmatic randomised controlled trial (ELVIS COVID-19)

**DOI:** 10.7189/jogh.14.05027

**Published:** 2024-12-13

**Authors:** Osman M Yusuf, Sandeep Ramalingam, John Norrie, Catriona Graham, Ahmad Kakakhail, Aimal T Rextin, Ramsha T Baig, Shahida O Yusuf, Bakhtawar Ahmad, Summan Zahra, Aziz Sheikh

**Affiliations:** 1 The Allergy & Asthma Institute, Islamabad, Pakistan; 2 National Health Service (NHS) Lothian, Edinburgh, UK; 3 Edinburgh Clinical Trials Unit, Usher Institute, University of Edinburgh, Edinburgh, UK; 4 Edinburgh Clinical Research Facility, University of Edinburgh, Edinburgh, UK; 5 National University of Modern Languages, Islamabad; 6 National University of Sciences and Technology (NUST), Islamabad; 7 Usher Institute, University of Edinburgh, Edinburgh, UK; 8 Nuffield Department of Primary Care Health Sciences, University of Oxford, Oxford, UK

## Abstract

**Background:**

In a previous pilot randomised controlled trial conducted on UK adults, we found that hypertonic saline nasal irrigation and gargling (HSNIG) reduced common cold symptoms, the need for over-the-counter medications, viral shedding, and the duration and transmission of the illness. It is unclear whether HSNIG improves outcomes of the coronavirus disease 2019 (COVID-19). Hypertonic saline can be prepared and HSNIG performed at home, making it a safe and scalable intervention, particularly well-suited for low- and middle-income countries.

**Methods:**

We conducted a pragmatic randomised controlled trial in Pakistan on adults with suspected or confirmed COVID-19, initially within 48 hours of symptom onset, later extended to within five days due to recruitment challenges. Participants were randomised to one of two groups: the intervention group received instructions on preparing a 2.6% hypertonic saline solution for HSNIG, while the control group was instructed on performing ablution for Muslim prayers (*wudu*), which involves nasal washing and gargling with tap water. Our primary outcome was the time to symptom resolution, measured by two consecutive days of scoring zero on relevant questions from the validated, self-reported, adapted short form of the Wisconsin Upper Respiratory Symptom Survey (WURSS-24). Secondary outcomes included the severity of all symptoms, the severity and time to resolution of individual symptoms, health care contacts (GP/physician, emergency contacts), hospital attendance (and length of stay if admitted), over-the-counter (OTC) medication (frequency and cost), and transmission to household contacts. The analysis was conducted on an intention-to-treat basis. Logistic regression was used to calculate adjusted odds ratios (aORs) of improvement and Cox regression to calculate adjusted hazard ratios (aHRs) for the time to improvement with accompanying 95% confidence intervals (CIs).

**Results:**

We randomised 576 people: 279 to the HSNIG group and 297 to the control group. Among those, 10 out of 279 (3.6%) in the HSNIG had symptom resolution, compared with 11 out of 297 (3.7%) in the control group (aOR = 1.20, 95% CI = 0.46– 3.22). The time-to-event analysis also showed no significant benefit (aHR = 1.23, 95% CI = 0.51–2.97). Excluding the 127 participants with no data on the primary outcome (who did not complete the study), 10 out of 222 (4.5%) in the HSNIG group had symptom resolution, compared to 11 out of 227 (4.8%) in the control group.

**Conclusions:**

HSNIG was not effective for individuals with suspected or confirmed COVID-19 who began the intervention within five days of symptoms onset and therefore cannot be recommended for use. Further investigation is needed for interventions started within 48 hours of illness onset.

**Registration:**

ClinicalTrials.gov (NCT05104372).

Coronavirus disease 2019 (COVID-19) continues to pose a major global health challenge [1,2]. It is caused by severe acute respiratory syndrome coronavirus 2 (SARS-CoV-2) [3], a highly transmissible RNA virus, which infects the nasopharyngeal tract through aerosols, droplets and fomites [4]. First discovered in December 2019, the viral genome has undergone mutations resulting in variants of concern with varying transmissibility and virulence [5–7]. There is growing evidence of the risk of infection even in fully vaccinated and booster-vaccinated individuals [8], highlighting the need for safe and effective treatments to limit the impact and spread of the virus.

Replication of SARS-CoV-2 begins in the upper respiratory tract (i.e. within the epithelial cells of lining the nasal passages) [9]. Nasal irrigation (i.e. flushing or washing of the nasal cavities with hypertonic salt solution) has long been used to treat upper respiratory tract symptoms such as rhinosinusitis [10]. Nasal irrigation has several potential benefits: it removes antigens, particulate matter, inflammatory factors, and extra mucus, while hydration helps improve the action of the respiratory cilia [11]. A meta-analysis showed that hypertonic saline solution irrigation is more effective than isotonic saline irrigation in treating sino-nasal diseases like rhinitis and rhinosinusitis [12]. In adults with a common cold, our Edinburgh and Lothians Viral Intervention Study (ELVIS) pilot randomised controlled trial (RCT) found that hypertonic saline nasal irrigation and gargling (HSNIG) effectively reduced the duration of illness by two days, decreased the use of over-the-counter (OTC) medication, reduced transmission within households, and led to a greater reduction in viral shedding [13].

Saline nasal wash was found to be effective for both acute and chronic upper respiratory tract infections (URTIs) and helped to reduce the severity of disease [14]. Additionally, regular saline nasal wash with seawater sprays has been shown to lead to a faster resolution of acute symptoms compared to a control group [15]. Compared to controls, significantly fewer individuals who used seawater spray prophylactically reported a URTI. There was also a significant decrease in the use of medications and related complications.

The benefit of nasal irrigation with hypertonic saline has usually been attributed to its ability to reduce the viscosity of the mucus layer. However, we have recently demonstrated a chloride ion-mediated antiviral effect in epithelial cells, which has a role in treating viral URTIs [16]. Epithelial cells use this innate antiviral effect as the first line of defence when they encounter DNA, RNA, enveloped, and non-enveloped viruses. We showed that this innate antiviral effect works against herpes simplex virus type 1, as well as respiratory viruses including respiratory syncytial virus, coxsackievirus B3, influenza A virus, and coronavirus 229E, which represent both enveloped and non-enveloped RNA viruses.

Post-hoc secondary analysis of data from the ELVIS pilot RCT in individuals recruited within 48 hours of onset of illness suggested that HSNIG reduced the duration of URTI caused by seasonal coronaviruses by an average of two-and-a-half days [17]. Although this subgroup analysis was small in size (n = 15), it suggested that HSNIG might offer a potentially safe, effective, and scalable intervention in those with COVID-19, particularly for those in resource-poor settings [17].

We conducted a RCT of HSNIG among newly diagnosed or suspected COVID-19 patients. The primary objective of the trial was to investigate whether HSNIG reduced the duration of symptoms in uncomplicated cases of COVID-19. Secondary objectives were to determine the effect of HSNIG on the severity of all symptoms, duration, and severity of individual symptoms and on OTC medication use.

## METHODS

### Study design and recruitment

We undertook a decentralised, pragmatic, web-based RCT of HSNIG vs nasal irrigation and gargling with water in adults aged 18 years and older with suspected or confirmed COVID-19. Due to the HSNIG or control being self-made at home by the participant, the study was not considered a Clinical Trial of an Investigational Medicinal Product.

In the initial phase of the trial, participants who were within the first 48 hours of developing any of the symptoms of COVID-19 were recruited through social medial channels. The symptoms included any respiratory symptoms, such as cough and shortness of breath, fever, muscle pain, headache, sore throat, new loss of taste or smell, and/or severe fatigue.

Following slow initial recruitment from 1 May to 15 October 2021 (n = 31, recruitment period 1), the recruitment process was reviewed. From 16 October 2021, eligibility was extended to five days from the start of any of the listed symptoms. New symptoms potentially associated with variants of concern were added, which included nausea and vomiting, diarrhoea, and congestion or runny nose. The recruitment process was modified to direct recruitment through trained recruiters inviting suspected patients visiting pharmacies, doctors' clinics, COVID-19 testing centres, and public welfare organisations working with COVID-19 patients. This provided 545 additional participants from 16 October to 22 November 2021 (recruitment period 2). Other than these changes, the same data collection protocols were used during the initial slow phase (recruitment period 1) and the much more rapid second phase (recruitment period 2) ensuring that the approach was consistent across the entire study.

### Eligibility and consent procedures

Participants needed to complete a baseline eligibility check online before being directed to a consent form. At the web portal, participants were provided with written information in the form of a patient information sheet and consent form. For those who needed further information, an email address (info@nasalwash.pk) was provided so they could have the opportunity to ask a member of the research team any question(s) they might have. There was no specified time given to the participant to consider the information, however, at consent, it was specified that they should be less than 48 hours (in recruitment period 1) or within five days (in recruitment period 2) of the onset of symptoms. We excluded participants below 18 years of age, pregnant women, immunosuppressed individuals (who were at higher risk of worsening their illness due to a poor protective immune response to a viral infection), as well as those unable to perform HSNIG for any reason, concurrently participating in another medical trial, unable to access Internet/email, living with another participant of this study, or those who were recommended for hospital admission by their clinician.

Consent was obtained electronically, with participants being given the option to consent to the future collection of samples for COVID-19 serological analysis. Of the participants who voluntarily consented to have their nasal swabs collected for testing for the presence of the SARS-CoV-2 virus, a small percentage were tested by reverse transcriptase-polymerase chain reaction. These participants were informed of government-approved laboratories in their cities, which provided the required testing facilities. Participants were asked to pay the costs of these additional tests upfront. The costs of the test and transport to the laboratory were reimbursed to the participant as soon as they sent the report of this test to the trial coordinating site either physically or electronically. Patients who had already had these tests done were excluded to avoid duplicate testing by reverse transcriptase-polymerase chain reaction, and their results were collected and recorded.

### Sample size calculations

Estimating an appropriate sample size for a web-based study in a rapidly evolving pandemic applicable to a resource-poor setting is very challenging. The ELVIS-PAKISTAN study was an implementation of the existing ELVIS-COVID study (in the UK) which was already struggling to recruit, and ELVIS-COVID itself was a reimagining of the ongoing ELVIS-KIDS study in children (and the pilot version in adults) with a common cold [13]. The original sample size calculation was based on a two-sample *t* test, considering a two-day reduction in time to symptom resolution with an assumed standard deviation of four days (85/randomised group), which after liberal adjustment for loss to follow-up (25%) and crossover from control to active (25%) indicated we needed to randomise 405 participants in total. We realised that, unlike the common cold and with the emergence of long COVID, an unknown number of people might not fully recover within 14 days. As a result, the primary outcome was switched to a time-to-event measure with censoring. An indicative sample size calculation using a log-rank statistic indicated that for an assumed 50% recovery rate on control, the study would have 90% power at a 5% level of significance to detect an improvement to 66%, which would equate to a hazard ratio (HR) of 0.60. Given that the requirement to achieve two reported consecutive days with no symptoms is a strict definition, a much lower resolution of symptoms at 10% would have 90% power at a 5% level of significance to detect a doubling of resolution to 20% (equivalent to an HR of 0.7).

### Randomisation and blinding

After obtaining their electronic consent, participants were centrally randomised to either the HSNIG (intervention) or *Wudu* (religious ablution control) groups, this being managed by the Edinburgh Clinical Trials Unit at the University of Edinburgh through a secure website, using a minimisation algorithm following Signorini [18] balancing on age (18–30, >30–50, >50 years) and sex.

Participants randomised to the intervention arm were directed towards a password-protected webpage featuring videos and instructions on how to prepare a 2.6% hypertonic saline solution and how to perform HSNIG.

Those randomised to the *Wudu* control arm were directed towards a password-protected webpage featuring a video and instructions on how to perform *Wudu* (which included nasal washing and gargling with tap water).

Due to the nature of the intervention, the participant could not be unaware of whether they were on HSNIG or control. Most of the primary and secondary outcomes were by participant self-report and hence could not be assessed blind to randomised allocation. The study database had queries completed unaware of the randomised allocations before finalising the locking of the trial database.

### Data collection

All pre- and post-randomisation data were collected through mobile phone/web-based questionnaires, comprising the Daily Study Diary, Daily Symptom reports, End of Illness questionnaires, and Day 14 questionnaire (Appendices 1–4 in the **Online Supplementary Document**). All participants were required to complete a daily symptom diary until day 14, and all participants were asked to complete the Day 14 questionnaire, which would document whether any other members of the participant’s household have developed symptoms of COVID-19. If a participant’s symptoms cleared before day 14, they would be invited to complete the End of Illness questionnaire (covering health care use, adverse events, acceptability, and infection in household contacts) on the day that these symptoms resolved, and continue to fill in the Daily Diary in case of recurrence of symptoms until day 14.

### Outcomes

The primary outcome was self-reported time to resolution (defined as two consecutive assessments of no symptoms) as assessed by completion of the validated, self-reported, UK-adapted short form of the Wisconsin Upper Respiratory Symptom Survey (WURSS-24) [19] completed daily. Secondary outcomes included the severity of all symptoms, the severity and time to resolution of individual symptoms, health care contacts (GP/physician, emergency contacts), hospital attendance (and length of stay if admitted), OTC medication (frequency and cost), and transmission to household contacts. The safety outcome was the number and type of adverse effects from performing HSNIG nasal washing and irrigation.

### Statistical analysis

We conducted all analyses according to the intention-to-treat principle, with *P* < 0.05 being considered statistically significant. There was no adjustment for any multiple comparisons (so caution is needed in interpreting the results of the many analyses). Categorical outcomes were compared using Fisher exact test.

When a ‘baseline’ measure is given, it refers to data collected on Day 1. The primary outcome, defined as the time to complete resolution of symptoms (scoring zero on two consecutive occasions on the validated self-reported UK-adapted short form of the WURSS-24), was analysed using a Cox proportional hazards model. This model included an indicator variable for treatment (HSNIG vs control) and was further adjusted for age (as a continuous measure), sex, recruitment period (before vs after 15 October 2021), and city (Islamabad, Rawalpindi, and other cities such as Gujranwala, Kohat, Lahore, Peshawar, and Sialkot). We also conducted sensitivity analyses for the primary outcome using logistic regression, which focussed solely on the occurrence of the event, without considering the timing of the event.

Secondary outcomes, comprising the severity of all and individual symptoms and time to improvement over day 1 of 2, 3, 4, or 5 points, were analysed using a mixed-effects repeated measures model. This model included participant as a random effect and treatment and visit as fixed effects. We did not include the interaction term of treatment by visit, as the purpose of this model was to estimate the average difference between the randomised groups throughout the intervention and not any evolution of that difference over time. We adjusted for the baseline analogue of the specific symptom being modelled, and sex, age and city. The time to resolution of each symptom was analysed using a Cox proportional hazards regression as per the primary outcome. We registered this trial with the ClinicalTrials.gov (NCT05104372).

### Deviations from original trial and analysis plan

As detailed above, the analysis of the primary outcome (time to complete resolution of symptoms) was changed from a comparison of mean times (linear model) to a time-to-event (Cox proportional hazards model) to accommodate those not achieving complete resolution within 14 days and withdrawals.

There was an intention to re-estimate the sample size after the first 100 randomised participants had mature data. However, due to the very slow recruitment of the first 31 participants over 6 months in the initial recruitment period, followed by the rapid recruitment of over 500 participants in five weeks during the second recruitment period, the opportunity to re-estimate the sample size was lost.

The second phase of recruitment was so rapid that we over-recruited to 576, exceeding the original target of 405. We did not formally assess the proportion of those randomised to the control group who crossed over to HSNIG (i.e. discovered how to use salt in the preparation of the washing fluid) given the self-reported nature of the trial data.

We reported our findings using the CONSORT checklist for controlled clinical trials (**Online Supplementary Document**).

## RESULTS

**Figure 1:**
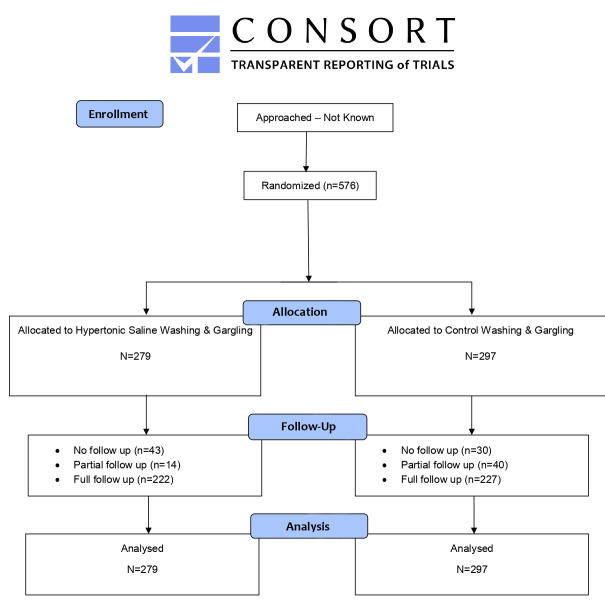
CONSORT flow diagram.

We recruited and randomised 576 people into this trial: 279 were randomised to the HSNIG intervention arm and 297 to the control arm (**Figure 1**, **Table 1**). As is common in studies relying on web-based recruitment with self-reported eligibility criteria, we also randomised some participants. Specifically, 116 (20.1%) participants reported having had symptoms for more than five days prior to randomisation: 73 (24.6%) in the control group and 43 (15.4%) in the intervention group. Additionally, 48 (8.3%) individuals were immunosuppressed, and 7 (1.2%) were pregnant.

**Table 1:** Baseline characteristics*

	Control (n = 297)	Intervention (n = 279)	Total (n = 576)
**COVID-19 details**			
COVID-19 confirmed by doctor	55 (18.5)	25 (9.0)	80 (13.9)
Told to go to hospital	87 (29.3)	63 (22.6)	150 (26.0)
Nasal swab to confirm COVID-19	72 (24.2)	48 (17.2)	120 (20.8)
*Of which tests positive?*	54 (75)	24 (50)	78 (65)
Other householder in study?	12 (4.0)	5 (1.8)	17 (3.0)
Self-isolation at home	81 (27.3)	52 (18.6)	133 (23.1)
Anyone in house with COVID-19 before you?	20 (6.7)	9 (3.2)	29 (5.1)
*Of which adults*, x̄ (SD); range	3.2 (1.5); 1–6	3.8 (1.2); 1–6	
*Of which children*, x̄ (SD); range	2.3 (1.4); 1–6	2.2(0.8); 1–3	
Anyone isolating with you?	18 (6.1)	10 (3.6)	28 (4.9)
*Of which adults*, x̄ (SD); range	1.9 (0.9); 1–4	2.3 (1.0); 1–4	
*Of which children*, x̄ (SD); range	1.4 (0.6); 1–3	3.0 (1.0); 2–4	
Individual ‘baseline’ score, x̄ (SD); range	4.30 (1.53); 1–7	3.96 (1.55); 1–7	
*Score of 1*	9 (3.6)	15 (6.0)	24 (4.8)
*Score of 2*	26 (10.5)	38 (15.4)	64 (12.9)
*Score of 3*	42 (17.0)	34 (13.7)	76 (15.3)
*Score of 4*	48 (18.4)	68 (27.3)	116 (23.4)
*Score of 5*	63 (25.5)	55 (22.1)	118 (23.8)
*Score of 6*	45 (18.2)	26 (10.4)	71 (14.3)
*Score of 7*	14 (5.7)	13 (5.2)	27 (5.4)
**Personal characteristics**			
Height in cm, x̄ (SD); range†	168 (10.4); 125–201	167 (10.8); 125–206	168 (10.6); 125–206
Weight in kg, x̄ (SD); range†	70.4 (14.4); 35–130	69.5 (14.7); 35–125	70.0 (14.5); 35–130
Gender (female)	113 (38.0)	96 (34.4)	209 (36.3)
Any symptoms >5 d	73 (24.6)	43 (15.4)	116 (20.1)
Health worker	14 (4.7)	9 (3.2)	23 (4.0)
Laboratory worker (micro/virology)	8 (2.7)	2 (0.7)	10 (1.7)
Weakened immune system	30 (10.1)	18 (6.5)	48 (8.3)
Pregnant	6 (2.0)	1 (0.4)	7 (1.2)
Smoke (current)‡	10 (11.4)	10 (13.5)	20 (12.4)
Vape (former)‡	7 (8.1)	3 (4.0)	10 (6.2)
Vape (current)‡	5 (5.8)	5 (6.7)	10 (6.2)
City			
*Gujranwala*	14 (4.7)	15 (5.4)	29 (5.0)
*Islamabad*	147 (49.5)	129 (46.2)	276 (47.9)
*Kohat*	4 (1.3)	8 (2.9)	12 (2.1)
*Lahore*	8 (2.7)	14 (5.0)	22 (3.8)
*Peshawar*	9 (3.0)	8 (2.9)	17 (3.0)
*Rawalpindi*	51 (17.2)	44 (15.8)	95 (16.5)
*Sialkot*	32 (10.8)	23 (8.2)	55 (9.5)
*Other*	32 (10.8)	38 (13.6)	70 (12.2)

SD – standard deviation, x̄ – mean

*Presented as n (%) unless specified otherwise.

†Missing 14 answers.

**‡**Missing 414 answers.

Self-reported adherence was good in both intervention and *Wudu* control arms, although the mean number of times of nasal irrigation and gargling was higher in the *Wudu* control arm (*P* < 0.0001) (**Table 2**). The mean times per day were 4.84 for *Wudu* control and 3.80 for intervention, with an adjusted difference of 1.03 (95% confidence interval (CI) = 0.91, 1.13) between the control and intervention groups. The distributions of the number of times nasal irrigation was done over the study period (control vs active) were 1–27 (13% vs 28%); 28–34 (22% vs 38%); 35–41 (32% vs 28%) and 42 or more (35% vs 6%).

**Table 2:** Estimated treatment effect (control vs intervention) for adherence and symptoms

Characteristic	Control, x̄ (SD)	Intervention, x̄ (SD)	Unadjusted difference (95% CI)*	*P*-value	Adjusted difference (95% CI)*	*P*-value
Adherence						
*Number of times gargled/d*	4.84 (1.93)	3.80 (1.11)	1.04 (0.94, 1.11)	<0.0001	1.03 (0.93, 1.13)	<0.0001
Symptoms (0–7)						
*How sick today?*	3.58 (1.58)	3.65 (1.66)	−0.07 (−0.16, 0.03)	0.16	−0.10 (−0.19, −0.01)	0.036
*Continuous cough*	2.40 (2.09)	3.03 (1.90)	−0.62 (−0.75, −0.50)	<0.0001	−0.67 (−0.78, 0.55)	<0.0001
*Shortness breath*	0.93 (1.52)	0.95 (1.58)	−0.02 (−0.12, 0.08)	0.69	−0.05 (−0.14, 0.04)	0.28
*Fever*	2.93 (1.86)	3.19 (1.85)	−0.26 (−0.37, −0.14)	<0.0001	−0.25 (−0.36, −0.15)	<0.0001
*Rigor with chills*	0.67 (1.30)	0.33 (0.94)	0.35 (0.28, 0.42)	<0.0001	0.33 (0.27, 0.39)	<0.0001
*Body/muscle pain*	1.66 (1.68)	1.30 (1.66)	0.35 (0.25, 0.45)	<0.0001	0.32 (0.22, 0.44)	<0.0001
*Headache*	1.63 (1.69)	1.23 (1.66)	0.40 (0.29, 0.50)	<0.0001	0.30 (0.20, 0.40)	<0.0001
*Sore throat*	1.40 (1.55)	1.18 (1.59)	0.21 (0.11, 0.31)	<0.0001	0.15 (0.05, 0.24)	0.0018
*Recent loss taste*	0.59 (1.31)	0.66 (1.49)	−0.07 (−0.15, 0.02)	0.13	−0.14 (−0.22, −0.06)	0.0008
*Recent loss smell*	0.66 (1.40)	0.78 (1.57)	−0.12 (−0.21, −0.03)	0.010	−0.10 (−0.19, −0.02)	0.016
*Runny nose*	1.85 (2.04)	2.46 (2.11)	−0.61 (−0.74, −0.48)	<0.0001	−0.68 (−0.80, −0.55)	<0.0001
*Blocked nose*	0.60 (1.23)	0.37 (1.10)	0.24 (0.16, 0.31)	<0.0001	0.21 (0.14, 0.28)	<0.0001
*Sneezing*	1.52 (1.78)	1.64 (1.92)	−0.12 (−0.24, −0.01)	0.034	−0.21 (−0.32, −0.10)	0.0002
*Scratchy throat*	1.15 (1.54)	1.13 (1.65)	0.02 (−0.08, 0.11)	0.76	0.02 (−0.08, 0.11)	0.73
*Hoarseness*	0.72 (1.36)	0.80 (1.51)	−0.08 (−0.17, 0.01)	0.080	−0.12 (−0.20, −0.03)	0.0059
*Head congestion*	0.76 (1.42)	0.83 (1.54)	−0.06 (−0.16, 0.03)	0.17	−0.12 (−0.21, 0.04)	0.0046
*Chest congestion*	0.80 (1.45)	0.87 (1.60)	−0.07 (−0.16, 0.03)	0.17	−0.14 (−0.23, −0.05)	0.0018
*Feeling feverish*	2.33 (2.14)	2.95 (2.03)	−0.61 (−0.74, −0.49)	<0.0001	−0.64 (−0.76, −0.51)	<0.0001
*General unease*	1.81 (1.83)	2.37 (1.80)	−0.56 (−0.67, −0.45)	<0.0001	−0.58 (−0.69, −0.46)	<0.0001
*Recent loss appetite*	0.81 (1.40)	0.86 (1.54)	−0.05 (−0.14, 0.05)	0.55	−0.09 (−0.18, −0.01)	0.038
*Diarrhoea*	0.44 (1.16)	0.21 (0.88)	0.23 (0.17, 0.30)	<0.0001	0.15 (0.09, 0.20)	<0.0001
*Vomiting/nausea*	0.51 (1.18)	0.18 (0.80)	0.33 (0.27, 0.39)	<0.0001	0.31 (0.26, 0.37)	<0.0001
*Dizziness*	0.56 (1.26)	0.25 (0.91)	0.32 (0.25, 0.39)	<0.0001	0.28 (0.22, 0.34)	<0.0001
*Stomach pain*	0.51 (1.22)	0.24 (0.93)	0.28 (0.21, 0.35)	<0.0001	0.26 (0.19, 0.32)	<0.0001
How severely was quality of life affected in the ability to						
*Think clearly*	1.32 (1.72)	1.36 (1.80)	−0.04 (−0.14, 0.07)	0.53	−0.05 (−0.15, 0.05)	0.34
*Sleep well*	2.29 (2.03)	3.08 (1.92)	−0.78 (−0.90, −0.66)	<0.0001	−0.79 (−0.91, −0.67)	<0.0001
*Breathe easily*	1.35 (1.75)	1.36 (1.82)	−0.01 (−0.11, 0.11)	0.93	−0.03 (−0.13, 0.08)	0.62
*Walk and climb stairs*	1.42 (1.86)	1.52 (2.04)	−0.10 (−0.22, −0.02)	0.12	−0.12 (−0.23, 0.00)	0.042
*Daily activities*	1.38 (1.80)	1.38 (1.82)	0.01 (−0.10, 0.12)	0.88	0.00 (−0.10, 0.10)	0.97
*Work outside home*	1.91 (2.25)	1.87 (2.21)	0.05 (−0.08, 0.18)	0.44	0.04 (−0.09, 0.17)	0.51
*Work inside home*	1.39 (1.87)	1.51 (2.07)	−0.11 (−0.23, 0.01)	0.081	−0.13 (−0.24, −0.02)	0.022
*Interact with others*	1.86 (2.23)	1.81 (2.20)	0.04 (−0.09, 0.18)	0.53	0.05 (−0.08, 0.18)	0.81
*Personal life*	1.34 (1.85)	1.44 (2.01)	−0.09 (−0.21, 0.03)	0.12	−0.11 (−0.23, 0.00)	0.043

CI – confidence interval, SD – standard deviation, x̄ – mean

*Control to intervention.

The proportion of individuals who reported two consecutive days of scoring zero on the question ‘How sick do you feel?’ (primary outcome) was low in both arms: 11 out of 297(3.7%) in the control group and 10 out of 279 (3.5%) in the intervention group (adjusted hazard ratio (aHR) = 1.23; 95% CI = 0.51, 2.97, *P* = 0.64) (**Table 2**). The sensitivity analysis using logistic regression gave similar results (adjusted odds ratio (aOR) = 1.20; 95% CI = 0.46, 3.22) (Table S1 in the **Online Supplementary Document**).

**Figure 2:**
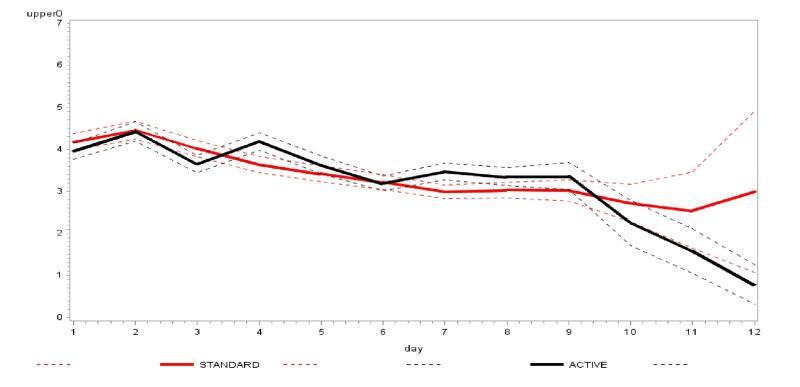
Primary outcome – score on ‘How sick today? (0–7)’ across time. Black solid line shows the active group (intervention, with dotted black lines representing lower and upper 95% confidence bands. The red solid line shows the standard group (control), with dotted red lines denoting the lower and upper 95% confidence bands.

**Figure 3:**
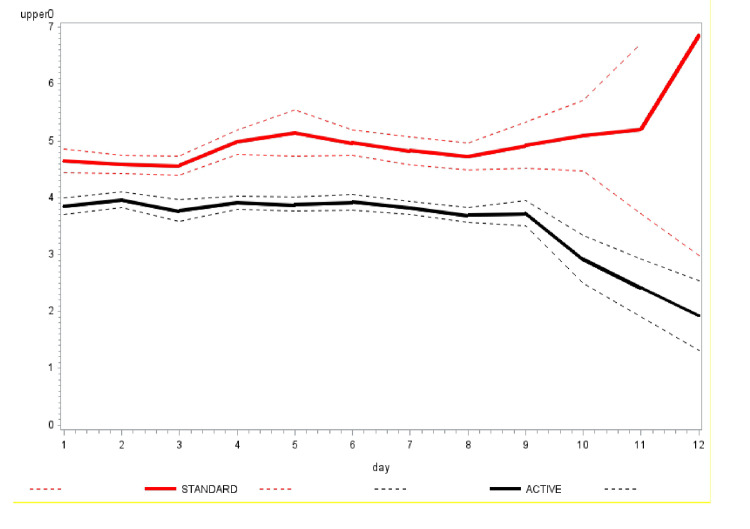
Average number of times nasal irrigation and gargling was performed daily. A black solid line shows the active group (intervention, with dotted black lines representing lower and upper 95% confidence bands. The red solid line shows the standard group (control), with dotted red lines denoting the lower and upper 95% confidence bands.

**Figure 4:**
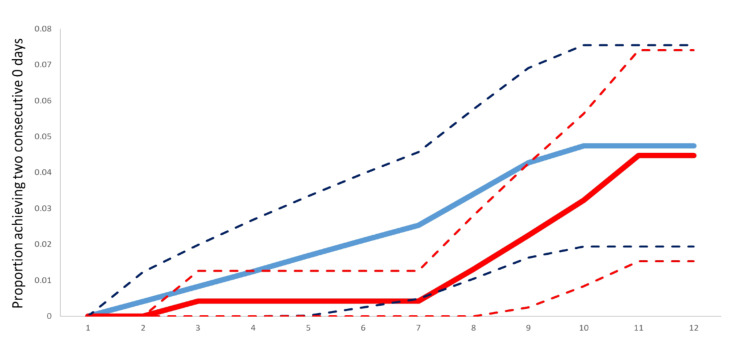
Kaplan-Meier Time to achieving two consecutive days zero score on ‘How sick do you feel’ (primary outcome). The red solid line shows the active group (intervention), while the red lines show the lower and upper 95% confidence bands. The blue solid line shows the standard group (control), with dotted blue lines showing the corresponding lower and upper 95% confidence bands. The x-axis shows the time in days in days.

The mean daily scores for the question ‘How sick do you feel today?’ were similar in both arms (**Figure 2**). The average number of times nasal irrigation and gargling were performed daily was consistently lower in the intervention arm compared to the control (**Figure 3**). The Kaplan-Meier curves of time to achieving two consecutive days of zero scores are shown in **Figure 4**.

We also conducted several *post-hoc* analyses on the primary outcome, varying the definition of resolution of symptoms, given the difficulty of defining this optimally. **Table 2** reports on varying the definition of the primary outcome by considering just one day with a score of zero (instead of two consecutive days as originally defined). It also includes analyses of improvement of two, three, four and five points over baseline at day one. Additionally, it explores relaxing the requirement of scoring zero on two consecutive days to alternative thresholds: either scoring zero or one, scoring zero, one, or two, and scoring zero, one, two or three. Interestingly, there was evidence of benefit for HSNIG over control for the definitions of time to the first improvement of four points (n = 38, 13% vs n = 56, 20% (aHR = 0.66; 95% CI = 0.44, 0.99, *P =* 0.047)), and five points (n = 9, 3% vs n = 23, 8% (aHR = 0.36; 95% CI = 0.17, 0.78, *P* = 0.0099)). However, when we varied the definition to two consecutive days scoring (zero, one, or two) instead of just zero, the advantage shifted to the control group (n = 85, 29% vs n = 51, 18% (aHR = 1.64, 95% CI = 1.64, 2.32, *P* = 0.005)). When extended to include a score of three (i.e. two consecutive days of scoring zero, one, two, or three) this advantage was maintained (n = 205, 69% vs n = 155, 56% (aHR = 1.43; 95% CI = 1.16, 1.77, *P *< 0.001)). Note that these were *post-hoc* analyses and multiple analyses need to be considered, but this does suggest that the definition of the primary outcome is critical and can make a difference to the direction and size of a treatment effect.

We were further concerned that the change in recruitment strategy may have influenced the findings. Specifically, the original plan was to recruit participants within two days of symptom onset, and that ran for the first 31 recruits (around six months to 15 October 2021) with much more rapid recruitment after that was relaxed to five days (545 recruits post 15 October 2021). There were nine successes in these 31, compared with 12 out of 545; and these split 3 out of 17 (control) vs 6 out of 14 (active) in the first 31 and 8 out of 280 (control) vs 4 out of 265 (active) in the following 545, giving non-significant treatment effects in different directions in the two periods (aHRs of around 0.3 and 2.0). Even though neither effect was statistically significantly different from no effect, a test of interaction was marginally statistically significant (*P*-value for interaction = 0.023) meaning that there was statistical evidence that these two estimated treatment effects (both statistically non-significant) were statistically significantly different between themselves. However, if we consider whether the participant was recruited within five days of symptom start rather than the recruitment period (first 31 vs next 545), we find that 116 participants had symptoms for more than five days, while 460 had symptoms for five days or less. In this analysis, there are 5 out of 73 (control) vs 6 out of 43 (active) with symptoms lasting more than five days (aHR ~ 0.5; 95% CI = 0.1, 2.0), and 6 out of 224 (control) vs 4 out of 236 (active) with symptoms lasting five days or less (aHR = 1.60; 95% CI = 0.4, 8.0). This shows no indication of a difference in treatment effect between those with symptoms lasting more than five days and those with symptoms lasting five days or less.

This was a pragmatic trial conducted during a pandemic, with partly internet-based recruitment, which limited our ability to thoroughly check all inclusion and exclusion criteria. As noted above, we identified 125 participants who breached an inclusion criterion; 116 had symptoms for more than five days, 48 had a weakened immune system, and 7 were pregnant.

There were also 78 who had a clinician/laboratory confirmed positive COVID-19 polymerase chain reaction test. There was a notable imbalance in the distribution between arms (54 control and 24 active) which we cannot explain. The resolution of symptoms was 0 out of 54 (0%) (control) vs 4 out of 24 (17%) (active) (odds ratio (OR) = 0.00; 95% CI = 0.00, 0.46, *P* = 0.0074). For those who were not COVID-19 positive, 11 out of 243 (control) vs 6 out of 255 (active) (OR = 1.97; 95% CI = 0.65, 6.58, *P* = 0.22).

The secondary outcome of severity of all symptoms was assessed using the ‘How Sick Today’ profile throughout the follow-up (**Table 3**), with scores ranging from 0 (lowest) to 7 (worst). The mean score for the control group was 3.58 (standard deviation (SD) = 1.58) compared to 3.65 (SD = 1.66) for the active group, resulting in an adjusted difference (control-active) of −0.10 (95% CI = −0.19, −0.01, *P* = 0.036). While this difference is statistically significant in favour of the control group, it is of no clinical importance.

**Table 3:** Primary outcome: response to question ‘How sick today (0–7)’ using Cox regression (time to event)

Outcome	Control, n (%)	Intervention, n (%)	HR (95% CI)	*P*-value	aHR (95% CI)	*P*-value
Primary						
*Two consecutive days scoring zero*	11 (3.7)	10 (3.6)	1.09 (0.46, 2.57)	0.84	1.23 (0.51, 2.97)	0.64
Supporting analyses						
*Time to one day at zero*	18 (6.1)	12 (4.3)	1.47 (0.71, 3.05)	0.30	1.83 (0.86, 3.88)	0.12
*Time to first improvement of 2 points*	179 (60.3)	168 (60.2)	0.96 (0.78, 1.19)	0.74	0.97 (0.71, 1.20)	0.79
*Time to first improvement of 3 points*	100 (33.7)	113 (40.5)	0.80 (0.61, 1.05)	0.11	0.81 (0.62, 1.06)	0.13
*Time to first improvement of 4 points*	38 (12.8)	56 (20.1)	0.64 (0.43, 0.97)	0.035	0.66 (0.44, 0.99)	0.047
*Time to first improvement of 5 points*	9 (3.0)	23 (8.3)	0.37 (0.17, 0.80)	0.012	0.36 (0.17, 0.78)	0.0099
*Time to two consecutive days scoring (zero or one)*	25 (8.4)	28 (10.0)	0.85 (0.50, 1.46)	0.56	0.85 (0.49, 1.47)	0.56
*Time to two consecutive days scoring (zero, one or two)*	85 (28.6)	51 (18.3)	1.60 (1.13, 2.27)	0.0078	1.64 (1.16, 2.32)	0.0050
*Time to two consecutive days scoring (zero, one, two or three)*	205 (69.0)	155 (55.6)	1.40 (1.14, 1.72)	0.0017	1.43 (1.16, 1.77)	0.0008

aHR – adjusted hazard ratio, CI – confidence interval, HR – hazard ratio

As for the severity of individual complications (the components of the WURSS-24), there were advantages for the control group over the active group in terms of a runny nose (which can be explained as hypertonic saline will increase nasal secretions for a while after the procedure), continuous cough, feeling feverish, and general unease (0.6), with differences of around 0.65 (95% CI = 0.5, 0.8) (**Table 3**). Conversely, headache, body/muscle pain, vomiting and nausea, dizziness, and rigour/chills were reported less frequently in the active group, with differences around 0.3 to 0.4 (95% CI of width around 0.2 units). The rest of the components showed statistically significant differences of less than 0.2 units in either direction (either favouring the active group or the control group), which were not considered clinically significant. For the quality-of-life dimensions, most components did not show any statistically significant difference (including thinking clearly, breathing easily, daily activities, activities outside home, and interaction with others), and a few showed borderline statistically significant effects which were of no clinical importance (walk and climb stairs, activities inside home, and personal life). The exception was sleeping well, which was 0.8 units lower on control than active (adjusted 95% CIs of width around 0.25).

The analogous time to resolution of individual symptoms and quality of life items showed similar patterns (data not shown, available on request).

In terms of health care resource uptake, there were no differences between the randomised groups. There was just one participant who visited their GP, and no hospital attendances. Concerning other treatments: 37 used herbal medications, 13 used exercise or yoga, and 26 used OTC medications, usually taken twice a day, with one-third using them at least three times a day. The OTC medications were mainly paracetamol, used for periods ranging from two to 14 days, and cost between 10 to 600 Pakistani rupees.

For household infections, there were no other members of households developing symptoms, and no adults or children were hospitalised. Among the participants with available data, 177 out of 496 (35.7%) reported developing further COVID symptoms after day one.

Data on side effects were only collected in the intervention group, reflecting the adaptation of the study from ELVIS COVID-19, which did not have a *Wudu* control arm. Among the participants in the control arm, 175 out of 248 (70.6%) reported pain or soreness in the nose, 216 out of 249 (86.8%) pain or soreness in the throat, 214 out of 249 (86.0%) a runny nose as a side effect, and 6 out of 249 (2.1%) an (unspecified) additional side effect. Of those with data, 199 out of 224 (88.8%) reported the intervention was difficult to do, 13 out of 224 (5.8%) stated they didn’t like it, 4 out of 224 (1.8%) felt there had not been an improvement, and 8 out of 224 (3.6%) attributed side effects directly to the intervention.

## DISCUSSION

We report the results of a large RCT of HSNIG in adults with suspected or confirmed COVID-19, which did not improve the resolution of symptoms or time to improvement when initiated up to five days after illness onset. As things stand, HSNIG cannot be recommended for use up to five days after symptom onset. Adequately powered high-quality randomised trials on this intervention early, within 48 hours of symptom onset, are needed.

The key strengths of this trial include its pragmatic, inclusive design, central randomisation, the use of a validated patient-reported outcome to assess our primary outcome, and the intention-to-treat approach used. Another strength is using a cheap and easy-to-prepare intervention that could easily be made at home. Finally, using an active control arm in which individuals were encouraged to rinse the nose and mouth, as is the case in *Wudu* rituals before daily prayers, was a culturally respectful approach that enabled us to assess the impact of using a hypertonic saline solution. This was considered more likely to be acceptable to the target, predominantly Muslim population of Pakistan than, for example, using a saline-based control.

Several limitations should be considered. First, we struggled with recruitment due to our primary reliance on a social media strategy-based approach and delays in presentation, and as a result, had to change strategy to allow a relaxation of the interval between the maximum duration of the symptom onset and recruitment from two to five days. This could have reduced the effectiveness of the HSNIG as the SARS-CoV-2 virus is known to move from the nasopharynx to the upper and lower respiratory tracts within the first few days of infection. Hence, there remains the need for a study on participants within 48 hours of symptom onset. A proportion of individuals were recruited even though they had exclusion criteria (having an illness onset of more than five days, being immunosuppressed, or being pregnant), although sensitivity analyses excluding such protocol violators did not suggest any major influence on the findings. These extensive *post-hoc* analyses raise the possibility of issues related to multiple testing. We chose not to formally adjust for multiple comparisons, since different readers may have varying questions of interest and differing philosophical perspectives on what constitutes an appropriate adjustment. Instead, as mentioned above, we have clearly stated that no adjustments for multiple comparisons have been made.

The distribution of clinically suspected and virologically confirmed COVID-19 cases appeared different at randomisation between arms. but this did not appear to excessively influence the reported findings. Future studies (e.g. in individuals with symptoms for less than 48 hours) should include virological confirmation at an early stage so that both arms identify similar numbers of infected individuals. Lastly, the addition of an active control arm brought its challenges. There is a possibility that the sample size which was originally calculated for the study without an active control arm could be underpowered to detect a difference with an active control. Our study did not extend to include testing the chlorine levels of the different tap waters used in different locations by the participants of the control group, so we were unable to assess the influence of chlorination on our study outcomes. Since tap water is chlorinated, there is a possibility that the chlorine in the water used as a control could reduce the difference between the two arms. In a recent study from Pakistan, coliform bacteria cultured from drinking water sources were resistant to chlorine (1 mg/L) [20]. Since bacteria take time to develop resistance, they should have been exposed for a long period of time to high doses of chlorine to be resistant to 1 mg/L of chlorine. Relatedly, nebulised hypertonic saline is used in the treatment of children with bronchiolitis. However, there is a debate on its use as some studies have not shown a significant effect compared to placebo. A recent meta-analysis of studies where normal saline was used as a placebo has shown a treatment effect in the placebo arm, which one would expect based on the antiviral effect mediated by chloride ions. Hence, consideration needs to be given to using alternative placebos (e.g. distilled water) [21].

Hypertonic saline solution was made at home and was thus not standardised. However, we produced a video and written materials in both Urdu and English, and provided pure salt, measuring cups, and spoons, which should have helped to reduce undue variation. We used this strategy as commercial hypertonic saline sprays are not easily available. It was also quick and easy to make the hypertonic saline solution and use the procedure as demonstrated in the ELVIS study on adults with the common cold [13]. However, the more significant illness associated with COVID-19 could have made the procedure more difficult in this population. Adherence was self-assessed, which may have posed a threat in an efficacy trial but is far less of a concern in a pragmatic effectiveness trial. Finally, it was not possible to blind individuals to the intervention and control arms thereby potentially introducing the risk of information bias. The study was adapted urgently from the ELVIS COVID-19 study in the UK, which did not have an active control. This may mean that the size of the difference we expected between the intervention and a non-active control may be smaller, and the impact of the control intervention compared to no intervention is not studied here. For a similar reason, the date of onset of illness was not included, which may have been helpful in determining if there was a benefit for people recruited within 48 hours of illness onset.

Blinding was difficult in the context of this pragmatic trial of HSNIG, and it is important to acknowledge that this may have introduced a major risk of bias. Any bias would likely have favoured the intervention, making it a crucial consideration if the trial results had been positive. However, given our null trial results, we think it is unlikely that the open nature of the trial had a major impact on the trial’s conclusions.

There is limited comparable trial-based data concerning HSNIG for the treatment of COVID-19. The one small trial conducted so far randomised SARS-CoV-2 infected subjects into one of three arms (n = 24 in each arm): either twice daily nasal irrigation with a topical detergent-based virucidal agent in hypertonic saline, hypertonic saline alone, or no intervention, showed no benefit from either treatment arm when compared to controls concerning the primary outcome measure of change in SARS-CoV-2 viral load over 21 days [22].

Future studies might benefit from considering an alternative primary outcome measure for symptom resolution, rather than using the WURSS-24 criterion of two consecutive days with a zero score, which is quite rigorous. Additionally, future research should aim to collect more objective data on adherence to the intervention and gather additional data on the severity, duration, and management of any potential HSNIG side effects.

## CONCLUSIONS

Although easily prepared and potentially scalable, this trial has found that HSNIG was ineffective in improving COVID-19 outcomes in adults in Pakistan with clinically suspected or confirmed COVID-19 when treatment was initiated within five days of illness onset. A further study incorporating the lessons learnt from the present study to determine the effectiveness of HSNIG started within 48 hours of illness onset with a longer follow-up period should be considered.

## Additional material


Online Supplementary Document
